# Acute and Long-Term Effects of Concurrent Resistance and Swimming Training on Swimming Performance

**DOI:** 10.3390/sports10030029

**Published:** 2022-02-24

**Authors:** Gavriil Arsoniadis, Petros Botonis, Gregory C. Bogdanis, Gerasimos Terzis, Argyris Toubekis

**Affiliations:** 1 Division of Aquatic Sports, School of Physical Education and Sports Science, National and Kapodistrian University of Athens, 17237 Dafne, Greece; garsoniadis@phed.uoa.gr (G.A.); pboton@phed.uoa.gr (P.B.); 2 Division of Sports Medicine and Biology of Exercise, School of Physical Education and Sports Science, National and Kapodistrian University of Athens, 17237 Dafne, Greece; 3 Sports Performance Laboratory, School of Physical Education and Sports Science, National and Kapodistrian University of Athens, 17237 Dafne, Greece; gbogdanis@phed.uoa.gr (G.C.B.); gterzis@phed.uoa.gr (G.T.)

**Keywords:** well-trained swimmers, dry-land training, endurance, training order

## Abstract

Dry-land resistance exercise (RT) is routinely applied concurrent to swimming (SWIM) training sessions in a year-round training plan. To date, the impact of the acute effect of RT on SWIM or SWIM on RT performance and the long-term RT-SWIM or SWIM-RT training outcome has received limited attention. The existing studies indicate that acute RT or SWIM training may temporarily decrease subsequent muscle function. Concurrent application of RT-SWIM or SWIM-RT may induce similar physiological alterations. Such alterations are dependent on the recovery duration between sessions. Considering the long-term effects of RT-SWIM, the limited existing data present improvements in front crawl swimming performance, dry-land upper and lower body maximum strength, and peak power in swim turn. Accordingly, SWIM-RT training order induces swimming performance improvements in front crawl and increments in maximum dry-land upper and lower body strength. Concurrent application of RT-SWIM or SWIM-RT training applied within a training day leads in similar performance gains after six to twelve weeks of training. The current review suggests that recovery duration between RT and SWIM is a predisposing factor that may determine the training outcome. Competitive swimmers may benefit after concurrent application with both training order scenarios during a training cycle.

## 1. Introduction

Competitive swimmers participate in dry-land training sessions aiming to improve several aspects of conditioning and increase in-water propulsive force [1,2,3]. During a dry-land session, swimmers may apply resistance training (RT) including various modes of exercise and specific apparatus [4,5,6]. Typically, RT may focus on muscular endurance with low external loads (3–4 sets, >12 repetitions, 40–60% of 1 repetition maximum (1-RM) [6,7,8,9] or maximum strength using high loads (3–5 sets, 3–5 repetitions, >85% of 1-RM, 2–3 min rest) [3,4,10,11,12]. Within a training microcycle, all dry-land sessions should be incorporated and adjusted according to swimming training sessions. Then, coaches should plan two or more RT sessions each week, prior to or following in-water swimming training (SWIM). Such a microcycle training plan is regularly repeated during a mesocycle or longer periods of training. There is evidence that long-term concurrent application of RT and SWIM training sessions may improve performance compared to SWIM training alone, and this has been extensively reviewed and supported with experimental findings [2,7,8,10,13,14]. However, several studies have indicated that concurrent training might pose a negative influence on various aspects of performance, and this is likely attributed to the distinct molecular responses activated following RT or endurance training [15,16].

Thus, in swimming training practice, the combination of RT and SWIM may create a conflicting environment within the skeletal muscle when it applies on the same day. Important factors that may determine the performance outcome is the content of training and the duration of recovery period provided between RT and SWIM sessions. For instance, compared to a maximum strength RT session, a muscular endurance RT session may cause a higher metabolic perturbation due to the short resting interval between sets and the higher volume of training [17,18,19,20,21]; whereas both RT training paradigms may induce similar decrements in phosphocreatine and glycogen stores [20,21,22,23]. Moreover, swimming training may include long-duration endurance and/or short-duration high-intensity bouts within a session in many cases combined with in-water RT [24,25]. Then, a variety of energetic, metabolic, and cardiovascular responses of a SWIM training session should be combined with neuromuscular and metabolic effects of the previous or subsequent RT training session, and vice versa [26,27,28].

Despite the growing evidence for a beneficial effect of RT on swimming performance, it is unclear whether RT should be applied prior or following a SWIM session. It has been recently reported that coaches prefer to apply SWIM before an RT session (SWIM-RT) [26], although it might be worthwhile pointing out that RT prior to SWIM (RT-SWIM) or RT only might also be applied depending on the coach’s preference. In both scenarios of concurrent resistance and swimming training, the recovery period between sessions may vary. This is because swimmers train in the water twice a day during long periods of their preparation. Then, a morning SWIM session may be followed by an RT session (SWIM-RT); however, one more SWIM session may also be applied a few hours later (RT-SWIM).

Despite the routinely applied practice of the RT-SWIM or SWIM-RT order within a training session, the combined acute and long-term effect of these training orders has received limited attention in swimming research. The purpose of the current review is to summarize the physiological responses and performance outcome following acute RT-SWIM or SWIM-RT applied within the same day and evaluate the subsequent long-term adaptations in well-trained swimmers.

## 2. Materials and Methods

A narrative review that examined the order effect of RT-SWIM or SWIM-RT in swimming performance was carried out. Two of the authors searched the databases Medline, Google scholar, and Sport Discus by September 2021, using the combinations of relevant keywords: “concurrent training” AND “order effect”, “concurrent training” AND “athletes”, “concurrent training” AND “swimming”, “concurrent training” AND “water polo“, “concurrent training” AND “acute effect” AND “athletes”, “concurrent training” AND “long-term effect” AND “athletes”. Moreover, the content of books of proceeding of the Biomechanics and Medicine Congresses from 1983 to 2018 were searched. 

**Figure 1:**
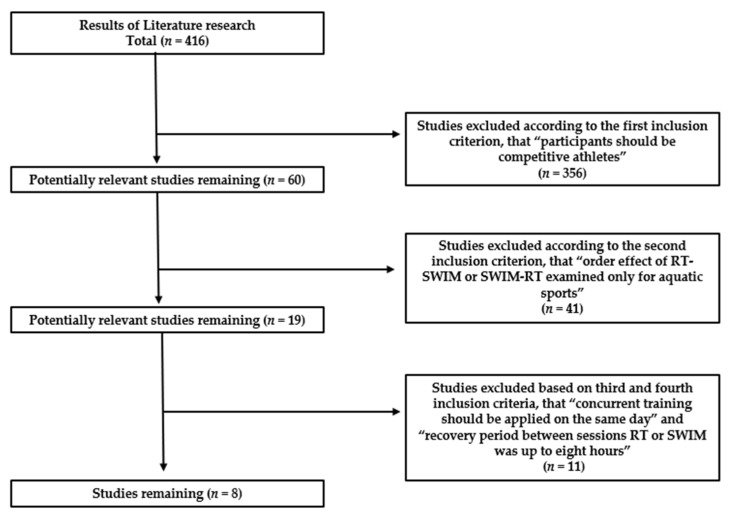
Flow chart of the literature search and studies used in the review according to inclusion criteria. RT: dry-land resistance training, SWIM: swimming training, *n* indicates the number of studies.

The searches identified 416 potentially relevant studies. Final selection was based on the following inclusion criteria, (a) participants should be competitive swimmers, (b) studies should clearly mentioned the RT-SWIM or SWIM-RT order in swimming or in other aquatic sport, (c) studies should refer that concurrent training of RT-SWIM or SWIM-RT order applied within the same day, and (d) the recovery period between the training sessions RT or SWIM was up to eight hours. The number and selections of studies included in this narrative review focusing on concurrent RT-SWIM or SWIM-RT training order and sports performance with physiological characteristics relevant to swimming and water polo are presented in Figure 1 and in Table 1. Moreover, the training level of swimmers that were included in the narrative review was classified according to previously suggested criteria [29] and is presented in Table 2.

## 3. Concurrent Resistance and Swimming Training: Acute Physiological Response and Performance Outcome in Swimmers

Two studies in swimming have examined the effect of RT and SWIM order on swimmer’s performance. To date, no study has examined the SWIM-RT order. In particular, these studies have used a repeated measures design and applied a counterbalanced [11] or randomized testing order [30]. Moreover, maximum strength (high loads) or muscular endurance (low loads) RT sessions were applied before a SWIM session that consisted of different training modes such as endurance [11] or repeated sprints [30]. Training characteristics of included studies in swimming that examined only the RT-SWIM order are reported in Table 3. 

### 3.1. Acute Effects of RT–SWIM

A recent study examined the effect of an RT session (3 sets, 3 repetitions, 85% of 1-RM, 4 min rest) on a subsequent endurance SWIM set of 5 × 400 m repetitions applied at a constant speed corresponding to 4 mmol·L^−1^ [11]. Performance during the 5 × 400 m was not affected by the RT-SWIM combination compared to that in the control condition (no RT before SWIM session). In addition, the force during a 10 s tethered swimming sprint that was performed 5 min before starting the 5 × 400 m set was no different compared to that in the control condition (no RT) [11]. Applying the RT session at high load (80% of 1-RM) 20 min before SWIM decreased performance in the best and mean time during 8 × 25 m repeated sprints [30]. The decrement in mean performance time was attenuated, while the best time was not affected following a low-load session (50% of 1-RM) [30]. However, it should be noted that the total volume of RT sessions was not equal (3 sets of 8 repetitions at 50% of 1-RM vs. 3 sets of 8 repetitions at 80% of 1-RM) allowing a likely better recovery following the low-load session.

The recovery period between RT and SWIM sessions varied from ~20 to ~40 min, and this may have affected metabolic restoration when concurrent training was applied in the same training unit. Despite the limited number of studies in swimming, it is likely that a 30 to 40 min recovery period between RT and SWIM allows adequate recovery of blood lactate concentration, heart rate, and oxygen uptake close to baseline before the start of a SWIM session [11]. However, blood lactate concentration was increased by 3% towards the end of the SWIM session when RT was preceding SWIM [11]. Possibly, maximum-strength RT enforced swimmers to a higher activation of anaerobic metabolism compared to that of the control condition (SWIM training only) aiming to maintain the required swimming speed during an endurance SWIM set [11]. It seems that a 30 to 40 min recovery interval between RT and SWIM sessions allows adequate removal of fatigue-related metabolites; any effect of an RT session, however, might be noticed later during the last part of a SWIM session, and it is likely attributed to neuromuscular fatigue [11,34,35]. The findings presented in studies applying acute RT-SWIM sessions suggest that when maximum strength dry-land RT is performed 20 min before a SWIM session, a decrement in best and mean performance time may occur during high-intensity swimming training [30]. On the contrary, when similar dry-land RT training was performed ~40 min prior to endurance training, there was no impact on swimmer’s endurance performance [11]. Coaches should be acknowledging the recovery period between RT and SWIM and the following SWIM session mode when it is applied concurrently on the same day.

### 3.2. Acute Effects of SWIM-RT

The search applied in the current review revealed the lack of studies testing the SWIM-RT order despite it being a likely approach during a real training setting. However, any homeostatic or metabolic and neuromuscular effect of the swimming session may affect efficiency, physiological responses in the following RT, and subsequent adaptations [26,36,37]. Interestingly, a SWIM training session (6 repetitions × 5 min front crawl) at an intensity 105% of critical speed (very heavy intensity domain) decreased maximal torque of shoulder internal rotators by ~23% [38] and a short-duration high-intensity SWIM of 4 × 50 m at extreme intensity domain (maximum effort) decreased isometric force and activation of shoulder muscles measured following 5 min of recovery [39]. Additionally, it is well-known that SWIM session duration and intensity may decrease muscle glycogen [40], affecting resynthesis of energy-providing substrates and performance in a subsequent high-intensity type of exercise [41]. A decrement of ~20% in average force production during maximal bilateral isometric force in endurance-trained athletes [42] and in back squat strength in resistance-trained athletes [43] was reported following 60 min steady-state running at 80% of maximum oxygen consumption preceding RT, compared to that with the reverse order. Considering the comparable findings between SWIM and running, it may suggest that intensity of a SWIM session may decrease maximum strength of swimmers due to neuromuscular fatigue [39,40,41,42,43]. Moreover, metabolic recovery is expected to affect subsequent performance, therefore, depending on the intensity and duration of SWIM training, heart rate, and blood lactate are expected to recover near to baseline values within 15–20 min [44]. Collectively, the above information indicates that swimmers starting an RT session following SWIM face decreased glycogen levels [40] and impaired muscle function [38,39]. In such a case, RT session performance and subsequent long-term adaptations may be compromised [45,46]. The overall effect of SWIM-RT may also be dependent on the duration of recovery between sessions, affecting restoration of physiological parameters. It should be pointed that long-term application of SWIM-RT training may induce adaptations both in skeletal muscle (i.e., increase muscle fibers area, capillary density) and in cardiovascular function (i.e., increase cardiac output and stroke volume) [15,16,47]. These potential adaptations may lead to a faster recovery rate between SWIM and RT sessions after a training period.

### 3.3. A Comparison of the Acute Training Orders

**Figure 2:**
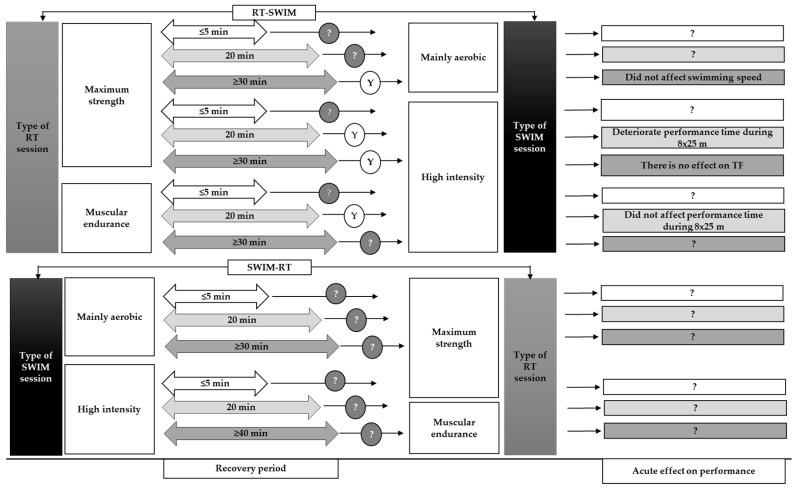
The lack of evidence for the comparison of the overall acute effect of RT-SWIM and SWIM-RT training orders on swimming performance. Data used from the swimming studies [11,30] that clearly reported the order and training content of RT-SWIM or SWIM-RT. Y: yes, it is suggested for training according to the reported performance effect, ?: remained undefined because there are no available data and RT-SWIM or SWIM-RT order and it is suggested for further research, TF: tethered swimming force.

There is no published data in swimming research directly comparing SWIM-RT and RT-SWIM acute effects. In other sports paradigms, applying endurance exercise at moderate intensity (70% of maximum oxygen uptake) before RT following 10 min recovery between sessions results in a higher blood lactate concentration at the end of combined endurance-RT sessions compared with that of RT-endurance exercise in resistance-trained individuals [48,49]. In contrast, both blood lactate concentration and oxygen uptake increase when a resting period of 5 min is applied between RT-endurance training compared with that of the endurance-RT order [50]. However, when the resting period between RT-endurance and endurance-RT training orders was 15 min, similar blood lactate responses were observed [34]. Whatever the case, blood lactate response reflects the effect of the last session (RT or endurance) and may be used as an indicator of exercise intensity but has limited value to judge the effectiveness or recovery following RT and endurance orders. Considering the previously mentioned findings, we hypothesize that a SWIM session preceding an RT session in the same training day may induce higher metabolic response compared to that expected in a subsequent RT. The last evidence should be tested with competitive swimmers. The lack of information concerning swimming literature and the need for further research is illustrated in Figure 2.

## 4. Concurrent Resistance and Swimming Training: Long-Term Physiological Adaptations and Performance Outcome in Swimmers

Effects of concurrent RT and SWIM training on swimming performance have been recently reviewed [2,13]. It appears that various RT training contents have been applied in swimmers of different age spectrum, focusing on dry-land maximum strength, muscular endurance, or power training [13]. Similarly, the SWIM training content that was combined with the RT training in the studies examined in the present review and varies in volume and intensity [3,6,9,10,24,31], including water polo training [32,33]. Moreover, most of the studies in swimming that examined the concurrent effect of RT and SWIM training did not clearly mention the recovery period or the order between RT and SWIM sessions [3,6,9,10,12,24]. Therefore, the conclusions presented in recently published reviews are based on findings obtained by a discrete evaluation of only one of the two combinations without any comment on the order of RT and SWIM sessions [2,13]. A detailed description of RT and SWIM training content of the studies that meet the criteria for inclusion in our analysis is presented in Table 4 and highlights the absence of intervention studies examining the long-term effect of RT-SWIM versus SWIM-RT order in competitive swimmers.

### 4.1. Long-Term Effects of RT–SWIM

Concurrent RT and SWIM applied in the same day may improve swimming performance more than swimming training per se [2,13]. Indeed, the order RT-SWIM has been reported in only two studies [7,8]. Amaro et al. [7] applied RT training focusing on upper and lower limb strength in adolescent swimmers for a six-week period. RT was applied 10 min prior to SWIM training and at the end of the intervention period, the swimmers showed 3.3% (0.7 s) improvement in 50 m sprint swimming performance compared to that of the control group [7]. Similar improvements in 25 m (4%; 0.6 s) and 50 m (2%; 0.6 s) sprint swimming performance, compared to that of the control group, were reported in post-pubertal competitive swimmers following six weeks of low-volume high-intensity RT training focusing only on upper limb strength and applied four times per week prior to swimming training, (Table 4; ref. [8]). Likewise, competitive swimmers improved their tethered force by 4% [7] when RT-SWIM training was applied concurrently on the same day in a six-week period [7]. Regarding strength level, increased maximal arm extension force by 13% and rate of force development during a bench press test by 25%, compared to those in the control group, were reported following concurrent RT-SWIM training for a six-week period [8]. RT-SWIM applied in water polo players during eight weeks of training improved 1-RM in a bench press test by 14–19% [32]. 

Alongside the strength improvement in the above-mentioned studies, RT-SWIM seems to improve both aerobic and anaerobic abilities. Specifically, in a group of well-trained water polo players, concurrent training involving the application of RT prior to specific water polo training for a period of eight weeks resulted in significant improvements in repeated sprint performance [32] as well as in swimming speed corresponding to 4, 5, and 10 mmol·L^−1^ [32]. Although tactical and technical in-water training of water polo players was of varied intensity and applied after RT, it did not interfere with strength gains [32,33]. Interestingly, in both studies, high-intensity aerobic training was applied on the previous or subsequent day, possibly facilitating improvement on repeated sprint performance and endurance indices [32,33].

Enhancement in performance and strength reported in the previous paragraphs is attributed to improvements in physiological [4,51] and neuromuscular parameters [51,52,53]. Specifically, performance gains may be attributed to improvements in maximal oxygen uptake that has been reported in top level cyclists, national level rowers, and well-trained runners after 6–12 weeks of concurrent RT-endurance training [54,55,56,57,58]. We may hypothesize that comparable enhancements in swimming performance may be attributed to improvement of both aerobic endurance and strength indices [32]. Likewise, strength gains may relate with improvements in motor unit recruitment, firing frequency, musculotendinous stiffness, and intramuscular coordination after long-duration RT-endurance training [44,51,52]. We would expect that such improvement following RT-SWIM training would be transferred to in-water propulsive power, subsequently enhancing swimming performance [1]. 

### 4.2. Long-Term Effects of SWIM-RT

The long-term effect of SWIM-RT order on performance has been reported in two studies, in which competitive swimmers participated [4,31]. Limited evidence suggests that six weeks of SWIM-RT, including explosive actions, improved the peak power during the push-off part of the swim turn by 2.6% [4]. In addition, SWIM-RT order improved swimmer’s 25 (3.0%; 0.3 s) and 400 m (3.8%; 9.7 s) performance, compared to that of the control group, when it was applied two times per week for a 12-week period [31]. It is interesting to note that only one study has directly compared concurrent endurance water-based and RT training, however, healthy females participated in the study, not allowing inclusion in the analysis of the present review [59]. Whatever the case, the water-based exercises used in endurance-RT order led to increments in 1-RM strength and muscle thickness similar to those with the RT-endurance order [59]. It seems that a SWIM-RT training order for a period of six weeks improves power required in explosive swimming actions such as the turns [4]. A longer-duration application of SWIM-RT for 12 weeks, improves short (25 m) and medium (400 m) distance swimming performance in competitive swimmers [31]. Despite SWIM-RT being a likely promising option, the evidence is limited, and further research is needed [4,31]. 

### 4.3. A Comparison of the Long-Term Training Orders

**Figure 3:**
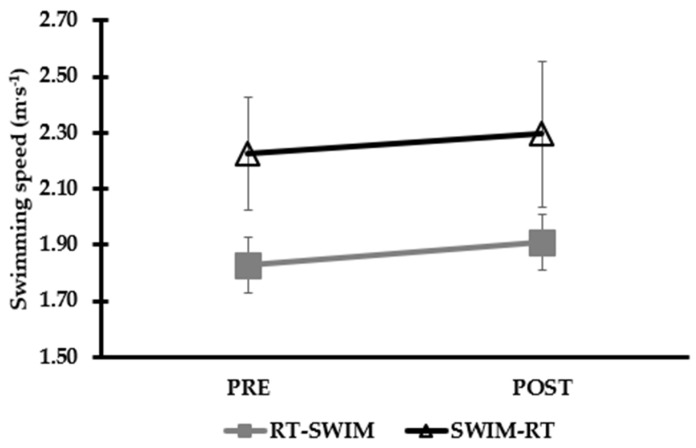
Changes in 25 m front crawl swimming performance after long-term concurrent resistance (RT) and swimming (SWIM) training (RT-SWIM) or SWIM-RT training. Data used from studies [8,31].

Irrespective of the order, RT-SWIM or SWIM-RT training, combined on the same day for a period of 6–12 weeks, two to four times per week, improves age-group swimmers’ performance in 25, 50, and 400 m front crawl (Table 4, Figure 3; refs. [7,8,31]). Similar findings in water polo suggested that RT-SWIM training for a period of eight weeks, two times per week, may improve water polo player’s sprint performance [32,33]. In addition, SWIM-RT training has a positive effect in swimmer’s peak power during the push phase of the swim turn [4]. Concerning the effect on strength, both training orders seem to increase 1-RM, but this finding was reported only in one study in water-based exercise with healthy female participants [59]. Provided that RT-SWIM or SWIM-RT orders have not directly tested in competitive swimmers, there is no supportive evidence of one over the other. Considering other sports paradigms (running, cycling, skiing) that compared RT-endurance or endurance-RT orders, there is evidence to suggest similar improvements in power output during high-intensity tests after 6–25 weeks [54,55,56,60]. In addition, similar maximal strength gains with both training orders after 8–14 weeks of training were observed [54,55,56,60,61]. Possibly, competitive swimmers will benefit from increased 1-RM strength in dry-land-based exercises (i.e., bench press) [8], or in-water-based tests [4], either with RT-SWIM or SWIM-RT orders. However, further research needs to be conducted. It should be recognized that within a swimming training session, not only endurance, but also other types of sprints or high-intensity training are applied concurrently. In such a scenario it is not only the endurance and RT order but also the sprint and RT orders that should be examined in future studies.

Other than the SWIM or RT training type, it seems that the recovery between sessions may affect long-term training adaptations. Despite a short recovery period between RT and SWIM sessions, swimming performance was improved (Table 4; refs. [7,8]). Likewise, a longer recovery period of 6–8 h, applied in cycling, running, or rowing, equally improved performance [62,63,64,65]. Recovery between sessions may depend on athletes’ level, individual characteristics, and the intensity or duration of the first session, whichever it is (SWIM or RT). Moreover, it may also depend on the location of the main set within each training session. For example, a high-intensity set in the beginning of the first session may have attenuated impact on the second session, and vice versa.

## 5. Conclusions

**Figure 4:**
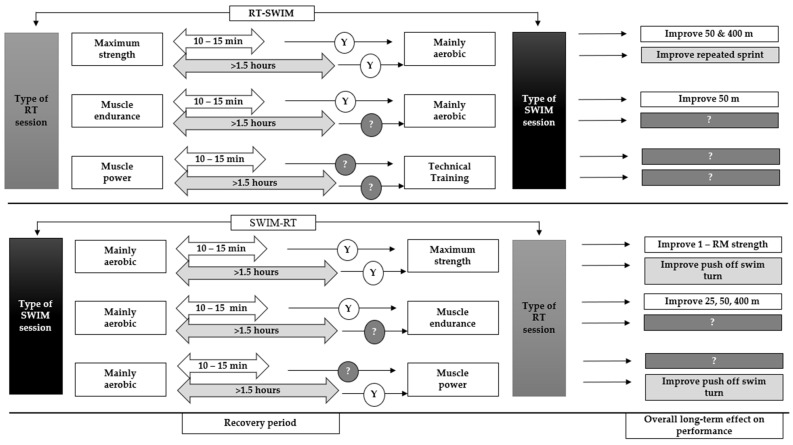
The overall long-term effect of RT-SWIM and SWIM-RT training orders on swimming performance. Data used from the swimming studies; [4,7,8,31,32,33] that clearly reported the order and training content of RT-SWIM or SWIM-RT. Y: yes, it is suggested for training according to the reported performance effect, ?: remained undefined because there are no available data and RT-SWIM or SWIM-RT order is suggested for further research.

Published research including competitive swimmers does not allow for a safe conclusion concerning the preferred or most effective order of acute concurrent training for performance enhancement in swimmers. A long duration (6–12 weeks, prevailing value: six weeks) of concurrent RT and SWIM training, independent of the applied order, seems to improve performance (Figure 4). The recovery duration between RT and SWIM or SWIM and RT training order as well as the training content of each RT and SWIM session are important key factors that should be acknowledged during a long-term concurrent application of both combinations. The current evidence indicates that a recovery period of 10–15 min using both training orders (RT-SWIM and SWIM-RT) is adequate to facilitate performance improvements following a period of training [7,8,31]. Moreover, when RT-SWIM or SWIM-RT are applied on the same day with a long recovery period between sessions (i.e., ~7 h), repeated sprints performance (i.e., 8 × 20 m) [32] and maximum strength are improved [4,8,32,33]. Furthermore, it seems that RT training planned to improve maximum strength or muscle endurance and SWIM training aiming to increase aerobic potential, when applied concurrently, do not show any interference effect phenomenon [28] and may be applied in training for competitive swimmers (Figure 4). There is only one study to our knowledge that directly compared RT-SWIM or SWIM-RT training orders, presenting increased muscle thickness and strength with RT-endurance after 12 weeks of concurrent application of water-based exercise [59]. However, in that study, RT was applied with in-water exercise, and SWIM included various aerobic type in-water activities [59]. The majority of the studies included highly trained swimmers with differences in swimming distance or stroke specialty and non-elite athletes. Further research is needed concerning the order of concurrent training, encompassing and categorized by stroke or distance specialty elite-level swimmers for safer conclusions.

Studies in individual sports such as running or cycling indicated similar improvements in performance during high-intensity tests and similar physiological adaptations with both training orders after a long-term period of training. It seems that RT-endurance or endurance-RT lead to similar performance gains or physiological responses after 12–16 weeks of training. Presumably, similar performance improvements and physiological outcomes may also occur in competitive swimmers.

It should be recognized that because of the small number of relevant studies examining the acute or long-term effects of both orders, the presented conclusion is subjected to several limitations. The competitive level (highly trained), age (pre-, post-pubertal), stroke specialty (front crawl, backstroke, breaststroke, butterfly), distance specialty (short or long) is very wide, and no data for competitive female participants are available. Moreover, the content of SWIM and RT training may present substantial differences and may focus on improvement of various abilities in or out of the water. Then, the acute and long-term performance and physiological adaptation may present huge diversity. All limitations should be considered by sport scientists and coaches when planning the short- and long-term preparation of swimmers aiming to improve competitive performance.

## 6. Practical Applications and Perspectives

Based on current evidence, we may suggest that a training session including as a first option either RT or SWIM may temporarily decrease performance in the second session. Coaches may use both options considering the main purpose of each session. The last needs to be examined in future studies with well-trained competitive swimmers. Concerning the long-term effect, both RT-SWIM and SWIM-RT may present comparable physiological adaptations and appear to improve performance in a similar manner.

## Figures and Tables

**Table 1:** Studies included in the narrative review aiming to demonstrate the acute and long-term order effect of dry-land resistance (RT) and swimming (SWIM) training.

Time Effect	Swimming Studies	Water Polo Studies
Acute	[11]	[30]
Long-term	[4,7,8,31]	[32,33]
Number of included studies	5	3

**Table 2:** Classification of swimmer’s training level.

Studies	Athletes’ Level	Training Background in RT (Years)	Training Routine (Times/Week)	Competing Level
[11]	Highly- Trained	≥3	6	National
[30]	Highly- trained	≥6	≥6	National
[7]	Highly- trained	-	-	National
[8]	Highly- Trained	-	-	National
[32]	Highly- trained	≥5	≥6	National
[33]	Elite/ international	≥5	≥6	National/ International
[4]	Elite/ international	≥2	≥6	National/ International
[31]	Highly- trained	≥2	≥5	National

≥: greater or equal, -: undefined, RT: dry-land resistance training.

**Table 3:** Acute physiological and performance effects of concurrent resistance (RT) and swimming (SWIM) training order.

			RT Set Characteristics						
Studies	Participants	Day Time of Training	Number of Sets and Repetitions	Exercises	Set Duration	SWIM Content	Order of Training	Recovery Time between RT-SWIM	Findings
[11]	*n* = 12, M, Well-trained swimmers, 19 ± 2 y	Morning: RT and SWIM sessions	3 sets × 5 reps @ 85% 1-RM with 4 min rest	Bench press Seated pull rowing Half squat (90°)	~45 min	5 × 400 m front crawl swimming @ speed corresponding to 4 mmol·L^−1^	RT-SWIM	40 min	RT-SWIM vs. SWIM only: similar oxygen consumption and heart rate. Increased lactate responses during RT-SWIM
[30]	*n* = 9, M, Competitive water polo players, 22 ± 2 y	Not reported	3 sets × 8 reps @ 50% 1-RM with 2 min rest and 3 sets × 8 reps @ 80% 1-RM with 2 min rest	Bench press Leg press	20 min	8 × 25 m all out front crawl with 30 s rest interval	RT-SWIM	20 min	Best time: RT (50% 1-RM)-SWIM and SWIM only better vs. RT (80% 1-RM)-SWIM Mean time: No difference RT (50% 1-RM)-SWIM vs. RT (80% 1-RM)-SWIM. SWIM only better vs. RT (80% 1-RM)-SWIM

RT: dry-land resistance training, END: endurance, 1-RM: 1 repetition maximum, s: seconds, *n* indicates the sample size in each study, M: males, F: females, y: years.

**Table 4:** Long-term effect of concurrent resistance (RT) and swimming (SWIM) training order (RT-SWIM) or SWIM-RT on swimming performance.

Studies	Participants	Day Time of Training	RT Training	SWIM Training	Study Design	RT Training Sessions per Week	Training Duration (Weeks)	Order and Recovery between Sessions	Findings
[7]	*n* = 21, M, Competitive Swimmers, 13 ± 1 y	Not reported	Group 1 (sets × reps): Week 1–2: 3 sets × 6–10 reps Week 3–4: 3 sets × 10–14 reps Week 5–6: 3 sets × 10–18 reps Group 2 (explosiveness): Week 1–2: 3 sets × 10–15 s Week 3–4: 3 sets × 15–20 s Week 5–6: 3 sets × 20–25 s Similar exercises in both groups: Medicine ball throws, jumps, dumbbell flys, Russian twists, push ups	Regular swimming training	Three groups repeated measures. Control and experimental	2	6	RT-SWIM Rec: 10 min	Group 1: RT-SWIM improved vertical jump (14%), ball throwing (7%) compared to control group. Group 2: RT-SWIM improved vertical jump (7%) ball throwing (17%) compared to control group. Fifty-meter swimming performance improved by ~3% compared to Group 1 and control group.
[8]	*n* = 19, M (N = 17), and F (*n* = 2), Competitive Swimmers Experimental group: 17 ± 1 y Control group: 18 ± 4 y	Not reported	3 sets × 3 reps @ 90% 1-RM with 5 min rest 2 sets × 2 reps @ 95% 1-RM with 5 min rest 1 set × 1 rep @ 100% 1-RM with 5 min rest	Regular swimming training	Two groups, repeated measures. Control and experimental	4	6	RT-SWIM Rec: Immediately after	RT-SWIM improved maximal arm extension force by 13%, rate of force development by 25%, 25 and 50 m performance by 4% and 2% respectively
[33]	*n* = 14, M, Elite water polo players Club 1: 30 ± 5 y Club 2: 29 ± 5 y	Morning: RT session Afternoon: SWIM session	4 sets × 4–5 reps @ 85–90% 1-RM with 3 min rest	Tactical and technical training after RT (same day). Next day 4 × 4 min or 16 × 100 m @ 106% of speed corresponding to 4 mmol·L^−1^ with 3 min rest	Two groups repeated measures. Control an experimental	2	8	RT-SWIM Rec: immediately after and after 24 h	RT-SWIM training improved swimming speed corresponding to 4, 5 and 10 mmol·L^−1^ by ~7–9% and 1-RM by ~14–19%
[32]	*n* = 8, M, Elite water polo players 27 ± 6 y	Morning: RT session Afternoon: SWIM session	4 sets × 4–5 reps @ 85–90% 1-RM with 3 min rest	Tactical and technical training after RT (same day) 4 × 4 min @ 106% of speed corresponding to 4 mmol·L^−1^ with 3 min rest (pre-season), 4–5 sets × 8 × 20 m maximum effort front crawl with 10 s rest (in season)	One group Cross-over design	2	8	RT-SWIM Rec: immediately after and after 24 h	RT-SWIM training improved repeated sprint swimming performance by 3.2% during pre-season compared to baseline period
[4]	*n* = 12, M (N = 10), and F (*n* = 2), Competitive Swimmers, 19 ± 1 y	Morning: SWIM and RT session	Group 1: 4–5 sets × 5–8 reps @85–90% 1-RM with 3–4 min rest Group 2: 4–5 sets × 3–5 reps @80–100% 1-RM with 2–3 min rest	Regular swimming training	Two groups repeated measures. No control group	3	6	SWIM-RT Rec: 1.5 h	SWIM-RT improved peak power during push off swim turn by 2.6%.
[31]	*n* = 10, M, highly trained collegiate swimmers, 20 ± 1 y	Afternoon: SWIM and RT session	3 sets × 8–12 reps	Intermittent swimming >85% of maximum oxygen uptake	Two groups repeated measures. No control group	2	12	SWIM-RT Rec: immediately after	SWIM-RT improved 25, and 400 m performance by ~4%

1-RM: 1 repetition maximum, min: minutes, Rec: recovery, *n* indicates the sample size in each study, M: males, F: females, y: years.

## Data Availability

Not applicable.

## References

[1] Barbosa T.M., Bragada J.A., Reis V.M., Marinho D.A., Carvalho C., Silva A.J. (2010). Energetics and biomechanics as determining factors of swimming performance: Updating the state of the art. J. Sci. Med. Sport..

[2] Crowley E., Harrison A.J., Lyons M. (2018). Dry-Land Resistance Training Practices of Elite Swimming Strength and Conditioning Coaches. J. Strength Cond. Res..

[3] Girold S., Jalab C., Bernard O., Carette P., Kemoun G., Dugué B. (2012). Dry-land strength training vs. electrical stimulation in sprint swimming performance. J. Strength Cond. Res..

[4] Jones J.V., Pyne D.B., Haff G.G., Newton R.U. (2018). Comparison of ballistic and strength training on swimming turn and dry-land leg extensor characteristics in elite swimmers. Int. J. Sports Sci. Coach..

[5] Roberts A.J., Termin B., Reilly M.F., Pendergast D.R. (1991). Effectiveness of biokinetic training on swimming performance in collegiate swimmers. J. Swim. Res..

[6] Tanaka H., Costill D.L., Thomas R., Fink W.J., Widrick J.J. (1993). Dry-land resistance training for competitive swimming. Med. Sci. Sports Exerc..

[7] Amaro N.M., Marinho D.A., Marques M.C., Batalha N.P., Morouço P.G. (2017). Effects of Dry-Land Strength and Conditioning Programs in Age Group Swimmers. J. Strength Cond. Res..

[8] Strass D., Ungerechts B., Wilke K., Resischle K. (1998). Effects of maximal strength training on sprint performance of competitive swimmers. Swimming Science.

[9] Weston M., Hibbs A.E., Thompson K.G., Spears I.R. (2015). Isolated core training improves sprint performance in national-level junior swimmers. Int. J. Sports Physiol. Perform..

[10] Aspenes S., Kjendlie P.L., Hoff J., Helgerud J. (2009). Combined strength and endurance training in competitive swimmers. J. Sports Sci. Med..

[11] Arsoniadis G.G., Bogdanis G.C., Terzis G., Toubekis A.G. (2020). Acute Resistance Exercise: Physiological and Biomechanical Alterations During a Subsequent Swim-Training Session. Int. J. Sports Physiol. Perform..

[12] Garrido N., Marinho D.A., Reis V.M., van den Tillar R., Costa A.M., Silva A.J., Marques M.C. (2010). Does combined dry land strength and aerobic training inhibit performance of young competitive swimmers?. J. Sports Sci. Med..

[13] Crowley E., Harrison A.J., Lyons M. (2017). The Impact of Resistance Training on Swimming Performance: A Systematic Review. Sports Med..

[14] Morouco P.G., Marinho D.A., Amaro N.M., Pérez-Turpin J.A., Marques M.C. (2012). Effects of dry-land strength training on swimming performance: A brief review. J. Hum. Sport Exerc..

[15] Fyfe J.J., Bishop D.J., Stepto N.K. (2014). Interference between concurrent resistance and endurance exercise: Molecular bases and the role of individual training variables. Sports Med..

[16] Fyfe J.J., Bartlett J.D., Hanson E.D., Stepto N.K., Bishop J.D. (2016). Endurance training intensity does not mediate interference to maximal lower-body strength gain during short-term concurrent Training. Front. Physiol..

[17] Aguiar S.S., Sousa C.V., Simões H.G., Neves R.V.P., Costa F., de Souza M.K., de Moraes M.R., Prestes J., Magalhães Sales M., Haro A.S. (2018). Acute metabolic responses following different resistance exercise protocols. Appl. Physiol. Nutr. Metab..

[18] Boone C.H., Hoffman J.R., Gonzalez A.M., Jajtner A.R., Townsend J.R., Baker K.M., Fukuda D.H., Stout J.R. (2016). Changes in Plasma Aldosterone and Electrolytes Following High-Volume and High-Intensity Resistance Exercise Protocols in Trained Men. J. Strength Cond. Res..

[19] Gotshalk L.A., Loebel C.C., Nindl B.C., Putukian M., Sebastianelli W.J., Newton R.U., Häkkinen K., Kraemer W.J. (1997). Hormonal responses of multiset versus single-set heavy-resistance exercise protocols. Can. J. Appl. Physiol..

[20] Howatson G., Brandon R., Hunter A.M. (2016). The Response to and Recovery from Maximum-Strength and -Power Training in Elite Track and Field Athletes. Int. J. Sports Physiol. Perform..

[21] Kang J., Hoffman J.R., Im J., Spiering B.A., Ratamess N.A., Rundell K.W., Nioka S., Cooper J., Chance B. (2005). Evaluation of physiological responses during recovery following three resistance exercise programs. J. Strength Cond. Res..

[22] Knuiman P., Hopman M.T., Mensink M. (2015). Glycogen availability and skeletal muscle adaptations with endurance and resistance exercise. Nutr. Metab..

[23] Tesch P.A., Colliander E.B., Kaiser P. (1986). Muscle metabolism during intense, heavy-resistance exercise. Eur. J. Appl. Physiol. Occup. Physiol..

[24] Girold S., Maurin D., Dugué B., Chatard J.C., Millet G. (2007). Effects of dry-land vs. resisted- and assisted-sprint exercises on swimming sprint performances. J. Strength Cond. Res..

[25] Valkoumas I., Gourgoulis V., Aggeloussis N., Antoniou P. (2020). The influence of an 11-week resisted swim training program on the inter-arm coordination in front crawl swimmers. Sports Biomech..

[26] Pollock S., Gaoua N., Johnston M.J., Cooke K., Girard O., Mileva K.N. (2019). Training Regimes and Recovery Monitoring Practices of Elite British Swimmers. J. Sports Sci. Med..

[27] Peyrebrune M.C., Toubekis A.G., Lakomy H.K., Nevill M.E. (2014). Estimating the energy contribution during single and repeated sprint swimming. Scand. J. Med. Sci. Sports..

[28] Vechin F.C., Conceição M.S., Telles G.D., Libardi C.A., Ugrinowitsch C. (2021). Interference Phenomenon with Concurrent Strength and High-Intensity Interval Training-Based Aerobic Training: An Updated Model. Sports Med..

[29] McKay K.A.A., Stellingwerff T., Smith E.S., Martin T.D., Mujika I., Goosey-Tolfrey V.L., Sheppard J., Burke L.M. (2022). Defining training and performance caliber: A participant classification framework. Int. J. Sports Physiol. Perf..

[30] Dalamitros A., Orologas P., Nousiou S., Semaltianou E., Zourladani A., Manou V. (2021). The acute effects of different resistance training loads on repeated sprint ability in water polo players. Hum. Mov. Sci..

[31] Trappe W.S., Pearson R.D. (1994). Effects of weight assisted dry-land strength training on swimming performance. J. Strength Cond. Res..

[32] Botonis P.G., Toubekis A.G., Terzis G.D., Geladas N.D., Platanou T.I. (2019). Effects of Concurrent Strength and High-Intensity Interval Training on Fitness and Match Performance in Water-Polo Players. J. Hum. Kinet..

[33] Botonis P.G., Toubekis A.G., Platanou T.I. (2016). Concurrent Strength and Interval Endurance Training in Elite Water Polo Players. J. Strength Cond. Res..

[34] Coffey V.G., Pilegaard H., Garnham A.P., O’Brien B.J., Hawley J.A. (2009). Consecutive bouts of diverse contractile activity alter acute responses in human skeletal muscle. J. Appl. Physiol..

[35] Doma K., Deakin G.B. (2013). The effects of strength training and endurance training order on running economy and performance. Appl. Physiol. Nutr. Metab..

[36] Greco C.C., Bassan M.N., Cesar T.E.A.S., Denadai B.S. Effect of an exhaustive swim exercise on isometric peak torque and stroke parameters. Proceedings of the XIIth International Symposium on Biomechanics and Medicine in Swimming.

[37] Dekerle J., King L. Fatigue of the shoulder’s internal rotators following a 200-m all-out swim. Proceedings of the XIIth International Symposium on Biomechanics and Medicine in Swimming.

[38] Dekerle J., Paterson J. (2016). Muscle Fatigue When Swimming Intermittently Above and Below Critical Speed. Int. J. Sports Physiol. Perform..

[39] Aujouannet Y.A., Bonifazi M., Hintzy F., Vuillerme N., Rouard A.H. (2006). Effects of a high-intensity swim test on kinematic parameters in high-level athletes. Appl. Physiol. Nutr. Metab..

[40] Costill D.L., Flynn M.G., Kirwan J.P., Houmard J.A., Mitchell J.B., Thomas R., Park S.H. (1988). Effects of repeated days of intensified training on muscle glycogen and swimming performance. Med. Sci. Sports. Exerc..

[41] Pascoe D.D., Gladden L.B. (1996). Muscle glycogen resynthesis after short term, high intensity exercise and resistance exercise. Sports Med..

[42] Taipale R.S., Schumann M., Mikkola J., Nyman K., Kyröläinen H., Nummela A., Häkkinen K. (2014). Acute neuromuscular and metabolic responses to combined strength and endurance loadings: The “order effect” in recreationally endurance trained runners. J. Sports Sci..

[43] Reed J.P., Schilling B.K., Murlasits Z. (2013). Acute neuromuscular and metabolic responses to concurrent endurance and resistance exercise. J. Strength Cond. Res..

[44] Bishop P.A., Jones E., Woods A.K. (2008). Recovery from training: A brief review: Brief review. J. Strength Cond. Res..

[45] Kraemer W.J., Patton J.F., Gordon S.E., Harman E.A., Deschenes M.R., Reynolds K., Triplett N.T., Dziados J.E. (1995). Compatibility of high-intensity strength and endurance training on hormonal and skeletal muscle adaptations. J. Appl. Physiol..

[46] Laursen P.B. (2010). Training for intense exercise performance: High-intensity or high-volume training?. Scand. J. Med. Sci. Sports..

[47] Sousa A.C., Neiva H.P., Izquiredo M., Alves A.R., Duarte-Mendes P., Ramalho A.G., Marques M.C., Marinho D.A. (2020). Concurrent training intensities A practical approach for program design. Strength Cond. J..

[48] Jones T.W., Howatson G., Russell M., French D.N. (2017). Effects of strength and endurance exercise order on endocrine responses to concurrent training. Eur. J. Sport. Sci..

[49] Jones T.W., Walshe I.H., Hamilton D.L., Howatson G., Russell M., Price O.J., Gibson A.S.C., French D.N. (2016). Signaling Responses After Varying Sequencing of Strength and Endurance Training in a Fed State. Int. J. Sports Physiol. Perform..

[50] Drummond M.J., Vehrs P.R., Schaalje G.B., Parcell A.C. (2005). Aerobic and resistance exercise sequence affects excess postexercise oxygen consumption. J. Strength Cond. Res..

[51] Berryman N., Mujika I., Bosquet L. (2020). Effects of Short-Term Concurrent Training Cessation on the Energy Cost of Running and Neuromuscular Performances in Middle-Distance Runners. Sports.

[52] Bell G.J., Petersen S.R., Wessel J., Bagnall K., Quinney H.A. (1991). Physiological adaptations to concurrent endurance training and low velocity resistance training. Int. J. Sports Med..

[53] Rønnestad B.R., Mujika I. (2014). Optimizing strength training for running and cycling endurance performance: A review. Scand. J. Med. Sci. Sports..

[54] Sedano S., Marín P.J., Cuadrado G., Redondo J.C. (2013). Concurrent training in elite male runners: The influence of strength versus muscular endurance training on performance outcomes. J. Strength Cond. Res..

[55] Rønnestad B.R., Hansen E.A., Raastad T. (2010). Effect of heavy strength training on thigh muscle cross-sectional area, performance determinants, and performance in well-trained cyclists. Eur. J. Appl. Physiol..

[56] Rønnestad B.R., Hansen E.A., Raastad T. (2011). Strength training improves 5-min all-out performance following 185 min of cycling. Scand. J. Med. Sci. Sports..

[57] Levin G.T., McGuigan M.R., Laursen P.B. (2009). Effect of concurrent resistance and endurance training on physiologic and performance parameters of well-trained endurance cyclists. J. Strength Cond. Res..

[58] Loturco I., Barbosa A.C., Nocentini R.K., Pereira L.A., Kobal R., Kitamura K., Abad C.C.C., Figueiredo P., Nakamura F.Y. (2016). A Correlational Analysis of Tethered Swimming, Swim Sprint Performance and Dry-land Power Assessments. Int. J. Sports Med..

[59] Pinto S.S., Cadore E.L., Alberton C.L., Zaffari P., Bagatini N.C., Baroni B.M., Radaelli R., Lanferdini F.J., Colado J.C., Pinto R.S. (2014). Effects of intra-session exercise sequence during water-based concurrent training. Int. J. Sports Med..

[60] Mikkola J.S., Rusko H.K., Nummela A.T., Paavolainen L.M., Häkkinen K. (2007). Concurrent endurance and explosive type strength training increases activation and fast force production of leg extensor muscles in endurance athletes. J. Strength Cond. Res..

[61] Taipale R.S., Mikkola J., Vesterinen V., Nummela A., Häkkinen K. (2013). Neuromuscular adaptations during combined strength and endurance training in endurance runners: Maximal versus explosive strength training or a mix of both. Eur. J. Appl. Physiol..

[62] Mikkola J., Rusko H., Nummela A., Pollari T., Häkkinen K. (2007). Concurrent endurance and explosive type strength training improves neuromuscular and anaerobic characteristics in young distance runners. Int. J. Sports Med..

[63] Young K.C., Kendall K.L., Patterson K.M., Pandya P.D., Fairman C.M., Smith S.W. (2014). Rowing performance, body composition, and bone mineral density outcomes in college-level rowers after a season of concurrent training. Int. J. Sports Physiol. Perform..

[64] Thiele D., Prieske O., Lesinski M., Granacher U. (2020). Effects of Equal Volume Heavy-Resistance Strength Training Versus Strength Endurance Training on Physical Fitness and Sport-Specific Performance in Young Elite Female Rowers. Front. Physiol..

[65] García-Pallarés J., Sánchez-Medina L., Carrasco L., Díaz A., Izquierdo M. (2009). Endurance and neuromuscular changes in world-class level kayakers during a periodized training cycle. Eur. J. Appl. Physiol..

